# Evidence-Based Management of MASLD: GRADE Evaluation of Pharmacological Therapies

**DOI:** 10.3390/ph19040605

**Published:** 2026-04-09

**Authors:** Eleni A. Karavia, Andreas Pittaras, Ourania Andreopoulou, Aikaterini Hatziri, Amalia Makrydimitri, Dimitrios Anagnostopoulos, Patroklos Vareltzis, Kyriakos E. Kypreos

**Affiliations:** 1 Pharmacology Laboratory, Department of Medicine, University of Patras, Rio Achaias, 26500 Patras, Greece; karaviae@hotmail.com (E.A.K.); andreaspittaras@gmail.com (A.P.); andreop@upatras.gr (O.A.); katerina_xatziri@hotmail.com (A.H.); 2 Adelco SA, 18346 Moschato, Greece; a.makrydimitri@adelco.gr (A.M.); d.anagnostopoulos@adelco.gr (D.A.); 3 Laboratory of Industrial Food Technologies and Agricultural Industries Technologies, Chemical Engineering Department, Faculty of Engineering, Aristotle University of Thessaloniki, 54124 Thessaloniki, Greece; pkvareltzis@cheng.auth.gr

**Keywords:** non-alcoholic fatty liver disease, metabolic dysfunction-associated liver disease, grading of recommendations assessment, development and evaluation, GRADE evaluation, medicines, evidence-based management

## Abstract

**Background/Objectives:** Non-alcoholic fatty liver disease (NAFLD), recently redefined as metabolic dysfunction-associated steatotic liver disease (MASLD), represents a growing global health burden closely linked to obesity, insulin resistance, and dietary patterns. Despite intense drug-development efforts, effective and widely approved pharmacological therapies remain limited. **Methods:** In this work, we systematically evaluated the quality of clinical evidence supporting currently proposed pharmacological treatments for MASLD/MASLD using the Grading of Recommendations Assessment, Development and Evaluation (GRADE) framework, focusing on phase III and IV clinical trials. **Results:** Our analysis demonstrates that overall quality of evidence for most pharmacological agents ranges from very low to moderate, primarily due to imprecision and suspected publication bias. **Conclusions:** Overall, our findings reinforce that, in the current therapeutic landscape, pharmacological therapies should be reserved for carefully selected patients and interpreted in the context of limited evidence certainty.

## 1. Introduction

Metabolic dysfunction-associated steatotic liver disease (MASLD), also termed non-alcoholic fatty liver disease (NAFLD) [1], represents the most prevalent chronic liver disorder worldwide [2,3]. The global prevalence of MASLD is estimated to exceed 25%, with particularly high rates in populations affected by obesity and type 2 diabetes mellitus [2].

MASLD encompasses a disease spectrum ranging from simple steatosis to metabolic dysfunction-associated steatohepatitis (MASH), progressive fibrosis, cirrhosis, and hepatocellular carcinoma [4,5]. The recent nomenclature switch from NAFLD to MASLD reflects a more precise alignment of the disease with its cardiometabolic underpinnings and aims to improve risk stratification and clinical management [1]. Given its strong association with metabolic syndrome and its potential progression to advanced liver disease, MASLD constitutes a major and growing public health challenge. Beyond hepatic complications, affected individuals carry increased cardiovascular risk, which remains the leading cause of mortality in this population [6,7,8].

Despite the magnitude of the health problem posed by MASLD, therapeutic options remain limited. Therapeutic lifestyle changes (TLCs)—particularly body weight reduction through dietary and behavioral interventions—constitute the basis of disease management. However, adherence to TLCs is often suboptimal, with many patients progressing to more advanced disease stages. Consequently, there has been intense interest in pharmacological therapies targeting metabolic pathways, inflammation, and fibrosis.

Recently, regulatory approvals have marked a pivotal milestone in the field. Resmetirom has received approval from both the U.S. Food and Drug Administration (FDA) and the European Medicines Agency (EMA) for the treatment of MASH with fibrosis [9,10]. In parallel, high-dose semaglutide has been granted accelerated approval by the FDA for patients with MASH and moderate to advanced fibrosis [11]. These developments have generated considerable optimism that disease-modifying pharmacotherapy may finally become available for selected patients.

Beyond these newly approved agents, several drugs traditionally used in MASLD management were originally developed for diabetes, dyslipidemia, or hypertension. Their use in MASLD has often been extrapolated from surrogate endpoints, small studies, or secondary analyses [1]. Even the recently approved therapies are supported primarily by histological surrogate outcomes and relatively short-term follow-up data.

The rapid expansion of therapeutic candidates and recent regulatory decisions therefore raise an important question: how robust is the clinical evidence underpinning these interventions? In this evolving landscape, clinicians, regulators, and guideline committees are required to make critical decisions in the context of heterogeneous—and at times uncertain—evidence.

Although multiple narrative reviews summarize pharmacological options for MASLD, few studies systematically appraise the quality and certainty of the underlying clinical evidence—beyond statistical significance—using a structured and internationally recognized methodology. The Grading of Recommendations Assessment, Development and Evaluation (GRADE) framework provides a transparent and reproducible approach for evaluating confidence in effect estimates across predefined clinical questions [12]. Importantly, GRADE distinguishes between apparent efficacy and the certainty of the supporting evidence—an essential distinction in a field where therapeutic urgency may outpace evidentiary robustness.

At this pivotal time—following recent regulatory approvals, ongoing late-phase trials, and increasing incorporation of pharmacotherapies into clinical practice—there is a clear need for a rigorous assessment of the strength and reliability of available evidence. A structured GRADE evaluation can clarify which therapies are supported by high-confidence data, which rely on moderate or low-certainty evidence, and where substantial research gaps persist.

The objective of the present study is therefore to systematically evaluate the quality of clinical evidence supporting pharmacological therapies used in MASLD, focusing on phase III and IV clinical trials. By applying the GRADE framework to predefined PICO questions, we aim to provide clinicians, researchers, and policy makers with a transparent assessment of current evidence certainty and to identify areas where further high-quality research is required.

## 2. Results

### 2.1. Thyromimetic Agent

#### Resmetirom

Resmetirom is an oral, liver-directed, thyroid hormone receptor beta-selective agonist for the treatment of MASH with liver fibrosis stages 1 and 2 [9,10]. In a phase III randomized, double-blind, placebo-controlled trial (MAESTRO-MASH), resmetirom demonstrated higher rates of MASH resolution and fibrosis improvement compared with placebo at 52 weeks [13]. Approximately 26–30% of treated patients achieved MASH resolution without worsening of fibrosis versus ~10% in the placebo group, while fibrosis improvement occurred in ~24–26% versus 14% with placebo [13].

GRADE assessment: Assessment of the quality of evidence was not possible as the conclusions rely on a single pivotal phase III trial with relatively short follow-up and surrogate histological endpoints. Although the trial was well designed and demonstrated statistically significant histological benefits, the evidence is limited by suspected publication bias and the lack of long-term outcome data.

### 2.2. Selective Inhibitor of Apoptosis Signal-Regulating Kinase 1

#### Selonsertib

Selonsertib, a selective inhibitor of apoptosis signal-regulating kinase 1 may reduce liver fibrosis in patients with nonalcoholic steatohepatitis and stage 2–3 fibrosis [14]. Two large phase III trials (STELLAR-3 and STELLAR-4) evaluated selonsertib in patients with advanced fibrosis or compensated cirrhosis [15]. Neither study demonstrated significant fibrosis improvement compared with placebo.

GRADE assessment: Very low quality of evidence. The evidence was downgraded due to risk of bias, imprecision, and strongly suspected publication bias. Despite adequate sample size, the lack of efficacy combined with methodological concerns and limited consistency across fibrosis stages resulted in very low confidence in effect estimates.

### 2.3. FXR Agonist

#### Obeticholic Acid

Obeticholic acid, a potent specific agonist of farnesoid X receptor (FXR), is currently indicated for the treatment of primary biliary cholangitis as monotherapy or in combination with ursodeoxycholic acid. The effects of obeticholic acid include regulation of bile acids, lipids, cholesterol, and glucose homeostasis [16]. In the REVERSE phase III trial in patients with compensated cirrhosis due to MASH, obeticholic acid showed only marginal differences in fibrosis improvement compared with placebo [17].

GRADE assessment: Assessment of the quality of evidence was not possible given that only one trial was performed. Clinical evidence from the REVERSE trial was primarily limited by risk of bias, a modest magnitude of clinical benefit, and reliance on surrogate histological endpoints without demonstrated long-term clinical outcomes.

### 2.4. Lipid-Lowering Therapy

#### Ezetimibe Monotherapy and Rosuvastatin/Ezetimibe Combination

Ezetimibe is a Niemann–Pick C1-like 1 (NPC1L1) protein inhibitor [18]. In a randomized, double-blind, placebo-controlled trial with 50 patients with biopsy-proven MASH who received either ezetimibe 10 mg orally daily or placebo for 24 weeks, it was reported that ezetimibe was not significantly different from placebo in reducing liver fat as measured by MRI–PDFF, leading to the conclusion that ezetimibe did not significantly reduce liver fat in MASH [19]. Another randomized, open-label trial enrolled 70 participants with ultrasound-confirmed MASLD to receive ezetimibe 10 mg plus rosuvastatin 5 mg or rosuvastatin monotherapy for 24 weeks. Combination therapy significantly reduced liver fat by MRI–PDFF, while fibrosis measured by MRE did not change significantly [20].

GRADE assessment: Assessment of the quality of evidence was not possible given that only one trial was performed per intervention. However, in the evaluation of the clinical evidence, that trial was subject to risk of bias, inconsistency, and imprecision, mainly due to small sample size, open-label design, and reliance on non-histological surrogate endpoints.

### 2.5. Incretin-Based Therapies

#### 2.5.1. Liraglutide

Liraglutide is among the first glucagon-like peptide-1 receptor agonists (GLP-1 RA) that were approved in 2010 for the treatment of T2DM. Among GLP-1 RAs, liraglutide is the most studied agent as a potential treatment option in MASLD, with multiple preclinical studies and clinical trials underlining its potential therapeutic implications in NAFL and MASH. This GLP-1 RA appears as an attractive treatment option in patients with MASLD, targeting various components of the metabolic syndrome, glucose intolerance, obesity and blood pressure that may be implicated in the pathogenesis of this disease [21]. In a randomized, open-label study where effects of liraglutide were evaluated on liver function and biomarkers of MASH in 30 obese Asian adults, it was reported that liraglutide was effective for decreasing hepatic steatosis and hepatocellular apoptosis in obese adults with MASLD, but benefits were not sustained after discontinuation, in contrast with lifestyle interventions [22].

GRADE assessment: Assessment of the quality of evidence was not possible given that only one trial was performed. However, the available clinical evidence from that trial was limited by risk of bias and imprecision, primarily due to limited sample size and short study duration.

#### 2.5.2. Semaglutide

Semaglutide is a more recent GLP-1 RA that has been already approved for the treatment of T2DM and obesity, while it seems an attractive therapeutic option for MASLD patients [23].

In a large phase III trial, semaglutide 2.4 mg achieved significantly higher rates of MASH resolution without worsening of fibrosis compared with placebo. However, fibrosis improvement was more modest.

GRADE assessment: Assessment of quality of evidence was not possible given that only one trial was performed. Although supported by a robust randomized design, the evidence suffered from suspected publication bias and dependence on surrogate histological endpoints, with long-term clinical outcome data still pending.

#### 2.5.3. Tirzepatide

Tirzepatide is a dual agonist of Glucose-Dependent Insulinotropic Polypeptide (GIP) receptor and GLP-1. A phase III substudy (SURPASS-3) showed significant reductions in liver fat content compared with insulin degludec in patients with type 2 diabetes [24]. However, histological endpoints were not assessed.

GRADE assessment: Assessment of the quality of evidence was not possible because only one trial was performed. However, the available clinical evidence from this trial had several limitations. Evidence from the SURPASS-3 trial was subject to multiple sources of bias, including indirectness (non-histological endpoints), inconsistency, risk of bias, and suspected publication bias, which limit confidence in extrapolation to fibrosis or clinical outcomes.

### 2.6. Sodium–Glucose Cotransporter-2 Inhibitors

#### 2.6.1. Empagliflozin

Empagliflozin is an SGLT-2 inhibitor approved for reducing the risk of cardiovascular death in adult patients with T2DM and cardiovascular disease [25]. Randomized trials demonstrated reductions in liver fat and improvement in liver enzymes compared with placebo, including studies in non-diabetic populations [26,27]

GRADE assessment: Moderate quality of evidence. The primary reason for downgrading was imprecision as studies were relatively small and primarily assessed surrogate imaging endpoints rather than histological fibrosis outcomes.

#### 2.6.2. Dapagliflozin

Dapagliflozin is one of the most recently developed SGLT-2 inhibitors, and many randomized controlled trials (RCTs) have evaluated the effect of dapagliflozin on improving the prognosis of MASLD in recent years [28]. A multicenter phase III trial reported significantly higher rates of MASH improvement and fibrosis benefit compared with placebo [29].

GRADE assessment: The study demonstrated robust randomized design and clinically meaningful histological endpoints without major methodological limitations. However, as only one phase III trial is available, no overall pooled GRADE assessment could be performed.

### 2.7. Insulin Sensitizers

#### 2.7.1. Pioglitazone

Pioglitazone, a thiazolidinedione is a synthetic ligand for peroxisome proliferator-activated receptors γ used as an adjunct to diet, exercise, and other antidiabetic medications to manage T2DM was assessed for its role in MASLD in several clinical trials [30]. Across three randomized trials, pioglitazone improved steatosis, inflammation, and, in some studies, fibrosis. Benefits were most consistent in patients with prediabetes or type 2 diabetes [29].

GRADE assessment: Moderate quality of evidence (overall). While individual trials showed methodological rigor, downgrading was applied due to imprecision and variability in fibrosis outcomes, particularly across diabetic and non-diabetic populations.

#### 2.7.2. Pioglitazone + Vitamin E

Combination therapy improved histological features and MASH resolution in one randomized trial but did not consistently improve fibrosis [31].

GRADE assessment: Very low quality of evidence. Downgrading was driven by risk of bias and imprecision as evidence is based on a single study with limited sample size.

#### 2.7.3. Metformin (±Vitamin E)

Metformin, a guanidine derivative, was discovered and used to treat diabetes as early as in the 1920s [32]. In the TONIC trial, metformin did not significantly improve sustained ALT reduction or broad histological outcomes compared with placebo [33].

GRADE assessment: Assessment of the quality of evidence was not possible because only one trial was performed. The clinical evidence from that trial was limited by imprecision, although the trial design itself was robust.

## 3. Discussion

The present work is novel in that it extends beyond a descriptive review of available phase III/IV clinical trials to critically appraise the quality of their evidence using a transparent, structured, and internationally validated methodological framework. We selected phase III and IV trials because they provide the highest level of clinical evidence relevant to regulatory approval, guideline development, and real-world therapeutic decision-making. It should be noted that this review does not aim at evaluating or comparing clinical guidelines from various health societies and organizations but rather focuses on objective evaluation of publicly available clinical trial data. Importantly, the present study was designed as a methodological appraisal of the certainty and quality of the available clinical evidence using the GRADE framework. It was not intended to develop clinical practice recommendations or to substitute for a formal guideline development process.

By applying the GRADE approach, we focus on the robustness and reliability of clinical evidence rather than the appraisal of the intrinsic pharmacological efficacy of individual agents. Although several pharmacological interventions demonstrate potentially favorable effects on histological or metabolic endpoints, substantial uncertainty remains regarding the strength, consistency, and generalizability of the supporting data.

The GRADE framework offers a structured and systematic method for evaluating the quality of evidence addressing a defined patient population, intervention, comparator, and outcome (PICO). While certain domains require informed judgment—such as the clinical relevance of outcomes or the interpretation of effect sizes—the majority of GRADE criteria are inherently objective, being grounded in trial design, methodological rigor, and reporting quality (Table 1). This robustness has facilitated the widespread international adoption of GRADE across medical disciplines and its endorsement by numerous scientific societies and regulatory authorities [12]. Our systematic assessment (Table 2) indicates that the quality of evidence supporting pharmacological agents currently used in MASLD management predominantly ranges from very low to moderate (Table 3). Low-quality evidence implies that further research is highly likely to substantially affect confidence in the estimated treatment effects and may lead to revised conclusions, while even moderate-quality evidence remains susceptible to meaningful change with additional well-designed studies.

The most frequent reason for downgrading was suspected publication bias, largely attributable to the limited number of phase III/IV trials, small sample sizes (often involving a few tens of participants), and frequent sponsorship by marketing authorization holders. Less commonly, downgrading resulted from risk of bias due to inadequate blinding or from imprecision related to insufficient information size, while inconsistency across studies was rarely observed (Table 2).

It should be noted that despite FDA authorization of resmetirom and high-dose semaglutide for the treatment of MASH with advanced fibrosis, the clinical evidence stems from one small trial of limited duration for each medicine, precluding PICO assessment for these two drugs. Apparently, more and longer-term clinical trials are needed to increase confidence in the clinical efficacy of these two agents. Collectively, our findings highlight significant challenges and limitations of MASLD pharmacotherapy. Therefore, these newer agents such as resmetirom or semaglutide should be used with caution and reserved for carefully selected patients with established MASH/MASH and medium to advanced fibrosis in whom disease progression confers substantial clinical risk. Nevertheless, intrinsic limitations in trial design, relatively small study populations, and short treatment durations restrict confidence in long-term clinical benefit. Larger trials with extended follow-up may alter the overall quality and certainty of evidence. The exclusion of ongoing trials, including those evaluating modern weight-loss medications, represents a limitation of our present evaluation; however, the completion of these studies may substantially influence future evidence assessments.

In contrast to patients with MASH and advanced fibrosis, individuals with early-stage MASLD (simple hepatic fat accumulation) are managed primarily through therapeutic lifestyle modifications. In such cases, dietary intervention is the cornerstone of patient management, consistently endorsed as first-line therapy [6,7]. Even when pharmacological treatment is considered, it is recommended only as an adjunct to lifestyle modifications. In the present work, however, we did not perform a GRADE evaluation of nutritional and lifestyle interventions because the available evidence is highly heterogeneous, encompassing diverse intervention types, study designs, active components, populations, and outcome measures [34]. Moreover, there is no established comparator and even widely cited dietary patterns, such as the Mediterranean diet, are not uniform but vary substantially across countries within the Mediterranean basin. This heterogeneity along with the limited number of large well-controlled randomized clinical trials and the frequent reliance on surrogate endpoints complicates the robust and meaningful GRADE assessment of nutritional interventions.

## 4. Methods

In this article, we evaluated the quality of clinical evidence for existing drugs against MASLD using the Grading of Recommendations Assessment, Development and Evaluation (GRADE) tool [35]. GRADE is a tool typically used to evaluate a body of evidence for a particular population/intervention/dcomparator/outcome also known as PICO. In our paper emphasis is placed here on the evaluation, that is, determining what the body of evidence tells us about the given PICO. Briefly, GRADE provides a methodology for classification of quality of evidence focusing on certain factors. These factors include: risk of bias (randomization, blindness, etc.), inconsistency (heterogeneity of population or intervention, etc.), indirectness (comparison to placebo, different target population, etc.), imprecision (optimal information size requirement, etc.) and publication bias (small number of trials, all of which are funded by industry, etc.) [35]. Evaluated drugs have been selected according to EASL–EASD–EASO Clinical Practice Guidelines on the management of MASLD [1].

**Figure 1:**
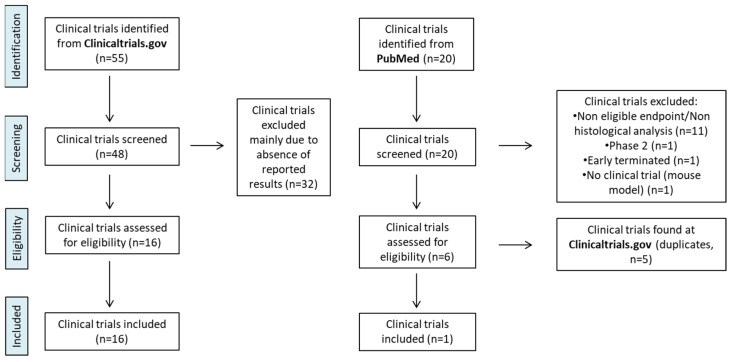
Strategy for collecting and evaluating clinical evidence for medicines of MASLD.

The ClinicalTrials.gov and PubMed databases were used to identify clinical studies using the following criteria: keywords “non—alcoholic fatty liver disease treatment” and “Name of each Drug”, “study results: with results”, “phase III”, “phase IV”. A total of 48 clinical trials were identified in ClinicalTrials.gov, of which 31 were excluded, mainly due to the absence of reported results or irrelevant endpoints (Figure 1, Supplemental Table S1). In addition, 20 clinical trials were identified in PubMed, of which 18 were excluded because they did not meet the primary endpoint or had already been identified through ClinicalTrials.gov (Figure 1, Supplemental Table S1). To apply GRADE to existing clinical evidence from different trials, the critical questions summarized in Table 1 were answered by YES or NO. This strategy allowed us to evaluate each study consistently and objectively and the results are listed in Table 2. Then, for each drug we proceeded to an overall evaluation of the body of relevant clinical evidence from all studies, according to the GRADE handbook https://gdt.gradepro.org/app/handbook/handbook.html accessed on 5 March 2026, and the results are listed in Table 3. Evaluation of clinical evidence for each PICO was performed using the academic version of GRADEpro tool (GRADEpro GDT: GRADEpro Guideline Development Tool [Software]. McMaster University and Evidence Prime, 2023. Available from gradepro.org.) The results of the Population, Intervention, Comparator, and Outcome (PICO) evaluations (where applicable) are presented in (Supplemental Tables S2–S4). In the absence of widely available approved pharmacological therapies with a formal indication for MASLD, we did not apply downgrading for indirectness in placebo-controlled trials as such an approach would have further reduced confidence in effect estimates without providing meaningful discrimination.

**Table 1:** Critical questions to evaluate the clinical evidence of each study according to GRADE methodology. CI stands for Confidence Interval.

Questions	Action
**Risk of Bias**	
Randomized	Yes = maintain, No = downgrade
Double blind	Yes = maintain, No = downgrade
Dropouts/withdrawals accounted for	Yes = maintain, No = downgrade
Large loses to follow-up	Yes = downgrade, No = maintain
All data taken into consideration	Yes = maintain, No = downgrade
Adherence to intention to treat analysis	Yes = maintain, No = downgrade
Stop early for benefit	Yes = downgrade, No = maintain
Failure to report outcomes	Yes = downgrade, No = maintain
**Inconsistency**	
Population heterogeneity	Yes = downgrade, No = maintain
Intervention heterogeneity	Yes = downgrade, No = maintain
Outcome heterogeneity	Yes = downgrade, No = maintain
**Indirectness**	
Indirect comparison	Yes = downgrade, No = maintain
Study population differs from target population	Yes = downgrade, No = maintain
Comparator differs among studies	Yes = downgrade, No = maintain
Study outcomes differ from outcomes of interest	Yes = downgrade, No = maintain
**Imprecision**	
Optimal information size	Yes = maintain, No = downgrade
Mean difference 95%CI includes 0	Yes = downgrade, No = maintain
Mean 95%CI ranges overlap	Yes = downgrade, No = maintain
Risk ratio 95%CI includes 1	Yes = downgrade, No = maintain
**Publication bias**	
Failure to report studies, especially those showing no effect	Yes = downgrade, No = maintain
Evidence arises from small trials funded by the drug company	Yes = downgrade, No = maintain

**Table 2:** Table summarizing the drugs, the phase III/IV clinical trials and the biases of each study stemming from the questions of Table 1.

Drugs for Metabolic Dysfunction-Associated Steatotic Liver Disease			
Name	ATC	Clinical Trial	Bias
Resmetirom	A05BA11	NCT03900429	Publication bias
Selonsertib		NCT03053050	Risk of bias, imprecision, publication bias
		NCT03053063	Risk of bias, imprecision, publication bias
Obeticholic acid	A05AA04	NCT03439254	Risk of bias
Rosuvastatin/ezetimibe combination	C10AA07/ C10AX09	NCT03434613	Risk of bias, inconsistency, imprecision
Liraglutide	A10BJ02	NCT02654665	Risk of bias, imprecision
Semaglutide	A10BJ02	NCT04822181	Publication bias
Empagliflozin	A10BK03	NCT04642261	Imprecision
		NCT02637973	Imprecision
Empagliflozin/pioglitazone combination	A10BK03/A10BG03	NCT05605158/NCT02686476	Risk of bias, inconsistency, imprecision
Dapagliflozin	A10BK01	NCT03723252	—
Pioglitazone	A10BG03	NCT00994682	Risk of bias
Pioglitazone	A10BG03	NCT00063622	Risk of bias, imprecision
Pioglitazone	A10BG03	NCT00227110	Risk of bias, imprecision
Pioglitazone/vitamin E combination	A10BG03/A11HA	NCT01002547	Risk of bias, imprecision
Metformin	A10BA02	NCT00063635	Imprecision
Tirzepatide	A10BJ07	NCT03882970	Risk of bias, inconsistency, indirectness, publication bias

O.A., A.H., P.V., A.M., performed the literature search independently, and E.A.K., A.P., D.A. and K.E.K. performed the independent appraisal of the collected manuscripts and GRADE evaluation of the clinical evidence. Disagreements were solved by consensus. All authors contributed to the drafting of the final submitted version of the manuscript. Critical questions used to evaluate the clinical evidence of each study are shown in Figure 1. The evaluation of clinical evidence for each medicine and individual trial is presented in Table 2. The overall assessment of confidence in the estimate of effect of phase III/IV clinical trials is presented in Table 3. As mandated by the principles of GRADE, only medicines with 2 or more trials were evaluated in Table 3.

**Table 3:** The overall assessment of the confidence in the estimate of effect of phase III/IV clinical trials. Overall assessment was performed only for drugs supported by ≥2 phase III/IV trials, in accordance with GRADE. Evaluations were performed with GRADEpro tool.

Drugs for NAFLD Comorbidities	No. of Studies	Limitations (Risk of Bias)	Indirectness	Inconsistency	Imprecision	Risk of Publication	Overall Assessment
Selonsertib	2	Serious	Not serious	Not serious	Serious	Strongly suspected	Very Low
Empagliflozin	2	Not serious	Not serious	Not serious	Very serious	Undetected	Low
Pioglitazone	3	Not serious	Not serious	Not serious	Very serious	Undetected	Moderate

## 5. Conclusions

The management of MASLD remains challenging, particularly in patients with progressive fibrosis. Although recent regulatory approvals mark an important milestone in the therapeutic landscape, the certainty of clinical evidence supporting pharmacological interventions varies considerably.

When evaluated using the GRADE framework and restricted to phase III/IV clinical trials, the overall confidence-in-effect estimates ranged predominantly from very low to moderate among agents supported by two or more trials. For example, regarding pioglitazone, which was evaluated across three phase III studies, the overall certainty of evidence was rated as moderate, primarily limited by imprecision. On the other hand, for resmetirom and high-dose semaglutide, conclusions must be interpreted cautiously. Although both agents demonstrated statistically significant histological improvements in large, well-designed phase III trials and have received regulatory authorization for specific MASH patients, each is currently supported by a single pivotal trial within the scope of our predefined inclusion criteria. In accordance with GRADE methodology, an overall certainty of evidence assessment cannot be performed when fewer than two trials are available. Therefore, the evidence supporting these agents should be considered promising but preliminary, pending confirmation from additional large independent phase III/IV studies and longer-term outcome data.

Similarly, several other pharmacological interventions demonstrated favorable effects on surrogate endpoints; however, limitations such as imprecision, risk of bias, indirectness, and suspected publication bias substantially reduced confidence in the estimated effects. Collectively, our findings highlight an important distinction between statistical efficacy and certainty of evidence. While multiple agents show therapeutic potential, the robustness, consistency, and long-term clinical relevance of these findings remain incompletely established for most drugs currently used in MASLD management.

Future large-scale, independently replicated, long-duration phase III/IV trials—ideally incorporating clinically meaningful endpoints beyond histological surrogates—are required to strengthen the evidence-base and enable high-certainty treatment recommendations.

Although the quality of evidence is central to evidence-based decision-making, daily clinical practice may not be determined by GRADE ratings alone. Additional considerations, including the benefit–risk balance, patient health condition, values and preferences, feasibility, and economic impact, also influence recommendations and introduce an unavoidable degree of subjectivity [12]. Therefore, until additional robust data become available, pharmacological therapy in MASLD should be applied judiciously, primarily in carefully selected patients with progressive disease and always in conjunction with structured lifestyle modification.

## Data Availability

Data is contained within the article or Supplementary Materials.

## Supplementary Materials

The following supporting information can be downloaded at: https://www.mdpi.com/article/10.3390/ph19040605/s1, Table S1: List of trials identified, Table S2: PICO analyses for empagliflozin, Table S3: PICO analyses for pioglitazone and Table S4: PICO analyses for selonsertib. References [35,36,37,38,39,40,41,42,43,44,45,46,47,48] are cited in the Supplementary Table S1.

## Abbreviations

The following abbreviations are used in this manuscript:

ALT	Alanine Aminotransferase
FXR	Farnesoid X Receptor
GLP-1 RA	Glucagon-Like Peptide-1 Receptor Agonist
GIP	Glucose-Dependent Insulinotropic Polypeptide
GRADE	Grading Of Recommendations Assessment, Development and Evaluation
HDL	High Density Lipoprotein
MASH	Metabolic Dysfunction-Associated Steatohepatitis
MASLD	Metabolic Dysfunction–Associated Steatotic Liver Disease
NAFLD	Non-Alcoholic Fatty Liver Disease
NPC1L1	Niemann–Pick C1-Like 1
MASH	Non-Alcoholic Steatohepatitis
NOAEL	No-Observed-Adverse-Effect Level
PICO	Population, Intervention, Comparator, and Outcome
RCTs	Randomized Controlled Trials
SGLT2	Sodium–Glucose Cotransporter 2
T2DM	Type 2 Diabetes Mellitus
