# Supplementary material for: Evidence-Based Management of MASLD: GRADE Evaluation of Pharmacological Therapies
Source: Pharmaceuticals (Basel). 2026 Apr 9;19(4):605. doi: 10.3390/ph19040605 (PMC13119189; doi:10.3390/ph19040605)
Supplement: Supplementary file 1 [file pharmaceuticals-19-00605-s001.zip › Supp Table S1.pdf]

Supplementary Table S1. List of trials identified. Those with green color had data publicly disclosed and met all requirements as described in materials and methods (included). The others did not have publicly reported results or endpoints were out of the scope of the present paper (DOI= Digital Object Identifier; NCT=number of clinical trial).

Active Pharmaceutical Ingredient	Clinical trials identified (clinicaltrials.gov)	DOI / NCT (Pubmed)	Reference
**Resmetirom**	NCT03900429	10.1056/NEJMoa2309000 / NCT03900429	[13]
		10.1038/s41591-023-02603-1 / NCT04197479	[36]
		10.1002/hep4.1657	[37]
		10.1111/apt.70382 / NCT03900429	[38]
**Selonsertib**	NCT03053050	10.1016/j.jhep.2020.02.027NCT03053050 / NCT03053063	[15]
	NCT03053063		
**Ursodeoxycholic acid**	NCT04910178	10.4166/kjg.2014.64.1.31	[39]
	NCT04977661		
**Obeticholic acid**	NCT02548351	10.1016/S0140-6736(19)33041-7 / NCT02548351	[40]
	NCT03439254	10.1016/j.cgh.2021.07.020.	[41]
**Omega-3 polyunsaturated fatty acids**	NCT01992809		
	NCT01277237		
	NCT01934777		
**Statins**	NCT07180745	10.1093/gastro/goac037	[42]
	NCT03434613		
	NCT05731596		
**Incretin mimetics**			
**Liraglutide**	NCT03068065		
	NCT02654665		
	NCT06501326		
	NCT02147925		
**Semaglutide**	NCT05195944	10.1056/NEJMoa2413258	[43]
	NCT04639414		
	NCT05813249		
	NCT04822181		

**Tirzepatide**	NCT07165028		
	NCT03882970		
**Sodium-glucose co-transporter-2 inhibitors**			
**Empagliflozin**	NCT02964715	10.1186/s12933-021-01237-2 / NCT03118336	[44]
	NCT04642261	10.2337/dc19-0641	[26]
	NCT07180745	10.1007/s00125-018-4702-3/	[46]
	NCT05942963		
	NCT04910178		
	NCT04976283		
	NCT04639414		
	NCT05147090		
	NCT02637973		
	NCT05605158		
**Dapagliflozin**	NCT05459701	10.1111/dom.15526	[45]
	NCT05308160	10.1007/s00125-018-4702-3	[46]
	NCT03723252		
	NCT06117137		
**Peroxisome proliferator-activated receptor agonists**			
**Pioglitazone**	NCT01002547	10.1056/NEJMoa0907929 / NCT00063622	[47]
	NCT00994682	10.1111/dom.15526	[45]
	NCT02365233		
	NCT00063622		
	NCT00227110		
**Saroglitazar**	NCT05872269		
	NCT04193982		
	NCT02265276		
**Metformin**	NCT00736385	10.1111/dom.15526	[45]
	NCT00063635	10.1053/j.gastro.2008.08.050	[48]
	NCT02365233		
