# Supplementary material for: Evidence-Based Management of MASLD: GRADE Evaluation of Pharmacological Therapies
Source: Pharmaceuticals (Basel). 2026 Apr 9;19(4):605. doi: 10.3390/ph19040605 (PMC13119189; doi:10.3390/ph19040605)
Supplement: Supplementary file 1 [file pharmaceuticals-19-00605-s001.zip › Supp Table S3.pdf]

Supplemental Table S3

Pioglitazone compared to placebo for NAFLD

Bibliography:

Certainty assessment							Summary of findings				
Participants (studies) Follow-up	Risk of bias	Inconsistency	Indirectness	Imprecision	Publication bias	Overall certainty of evidence	Study event rates (%)		Relative effect (95% CI)	Anticipated absolute effects	
							With placebo	With pioglitazone		Risk with placebo	Risk difference with pioglitazone
**New outcome**											
266 (3 RCTs)	not serious	not serious	not serious	very serious[a]	strong association	⨁⨁⨁◯ Moderate[a]	34/155 (21.9%)	89/111 (80.2%)	not estimable	34/155 (21.9%)	

CI: confidence interval

Explanations

a. Two out of three studies have no optimal size information requirement.
