# Supplementary material for: Evidence-Based Management of MASLD: GRADE Evaluation of Pharmacological Therapies
Source: Pharmaceuticals (Basel). 2026 Apr 9;19(4):605. doi: 10.3390/ph19040605 (PMC13119189; doi:10.3390/ph19040605)
Supplement: Supplementary file 1 [file pharmaceuticals-19-00605-s001.zip › Supp Table S4.pdf]

Supplemental Table S4

Selonsertib compared to placebo for NAFLD

Bibliography:

Certainty assessment							Summary of findings				
Participants (studies) Follow-up	Risk of bias	Inconsistency	Indirectness	Imprecision	Publication bias	Overall certainty of evidence	Study event rates (%)		Relative effect (95% CI)	Anticipated absolute effects	
							With placebo	With Selonsertib		Risk with placebo	Risk difference with Selonsertib
**New outcome**											
1007 (2 RCTs)	serious[a]	not serious	not serious	serious[b]	publication bias strongly suspected[c]	⨁◯◯◯ Very low[a,b,c]	26/331 (7.9%)	24/676 (3.6%)	not estimable	26/331 (7.9%)	

CI: confidence interval

Explanations

a. Both of studies are early terminated
b. Both of studies had no optimal size information requirment
c. Evidence arises from small trials funded by the company
